# Lithobius (Monotarsobius) meifengensis, a new species of centipede from high altitude forest in central Taiwan (Chilopoda, Lithobiomorpha, Lithobiidae)

**DOI:** 10.3897/zookeys.741.21036

**Published:** 2018-03-07

**Authors:** Jui-Lung Chao, Kwen-Shen Lee, Hsueh-Wen Chang

**Affiliations:** 1 Invertebrates Section, Zoology Department, National Museum of Natural Science,1 Guancian Road, Taichung, Taiwan 404, R.O.C; 2 Department of Biological Sciences, National Sun Yat-Sen University, 70 Lien-Hai Road, Kaohsiung, Taiwan 804, R.O.C.

**Keywords:** *Lithobius*, male secondary sexual character, taxonomy

## Abstract

Lithobius (Monotarsobius) meifengensis **sp. n.** occurring at Mei-Feng Farm, Highland Experimental Farm of National Taiwan University, Nantou, Taiwan, is characterised by a male secondary sexual character on leg 15, a very large ventral swelling occupying almost 50% of the ventral surface of the femur; the gently curved apical region bearing approximately 20 short setae and numerous very small pores of flexo-canal epidermal glands. This male secondary sexual character is described for the first time in the genus *Lithobius*.

## Introduction

In some male *Lithobius* species, there are secondary sexual characters on the dorsal surface of the femur or tibia of legs 14 and 15 (Lewis, 1981). In *Lithobius calcaratus* C. L. Koch, 1844, leg 15 has a dorsal wart-like projection on the inner end of the femur (Carballo et al. 1992). For four species described from East Asia: a characteristic crest with long setae as a Chasen-bamboo whisk at the distal end of the tibia of anal legs is present in Lithobius (Monotarsobius) tuberculatus (Murakami, 1965); a small oval region densely covered with small pores of epidermal glands and long setae at the dorsal tibia of legs 15 in Lithobius (Monotarsobius) sasanus (Murakami, 1965); a dorsal wart-like projection is present on the femur of legs 15 in Lithobius (Monotarsobius) dziadoszi Matic, 1970, and a dorsal wart-like projection on the tibia of legs 15 in Lithobius (Monotarsobius) riedeli Matic, 1970. Eason (1973) described the male secondary sexual characters of six species originally assigned to *Lithobius*, mostly from Central America. a shallow excavation bearing a tuft of setae on the dorsal surface of 14^th^ tibia, and a small wart-like outgrowth projection from the dorsal excavation on the 15^th^ tibia in *Vulcanbius godmani* (Pocock, 1895); a dorsal shallow excavation on the 14^th^ tibia in *Vulcanbius salvini* (Pocock, 1895); a crest rises from the dorsal excavation on 15^th^ tibia in *Vulcanbius vulcani* (Pocock, 1895); a dorsal wart-like projection on 15^th^ tibia in both *Guerrobius pontifex* (Pocock, 1895) and *Guerrobius humberti* (Pocock, 1895); a dorsal wart-like projection on 15^th^ femur in *Lithobius obscurus* (Meinert, 1872).

The subgenus Lithobius (Monotarsobius) is among the poorly studied taxa of East Asia (Takakuwa 1941a, b; Wang 1955, 1956, 1957, 1959, 1963; Murakami 1965; Matic 1970; Pei et al. 2011; Ma et al. 2009, 2012, 2014). Three species of Lithobius (Monotarsobius): Lithobius (Monotarsobius) holstii (Pocock, 1895), Lithobius (Monotarsobius) obtusus (Takakuwa, 1941), and Lithobius (Monotarsobius) ramulosus (Takakuwa, 1941), were recorded from Taiwan by Takakuwa (1941a, b) and Wang (1955, 1956, 1957, 1959, 1963). However, Takakuwa’s specimens were destroyed in an air attack during the war in 1945, and we could not locate Wang’s specimens in Taiwan. We studied specimens of centipedes collected from Taiwan, deposited at the National Museum of Natural Science (NMNS) and here describe a new lithobiid.

## Materials and methods

Forty-nine specimens of both sexes of the new species treated below were collected from Mei-Feng Farm, Highland Experimental Farm of National Taiwan University, Nantou, Taiwan. The material was studied using stereo-microscope and SEM. Type specimens are preserved in 75% alcohol and deposited in the department of Zoology, National Museum of Natural Science, Taichung, Taiwan. Terminology for external anatomy follows Bonato et al. (2010). The following abbreviations are used in the text and tables:

## Taxonomy

### Lithobius (Monotarsobius) meifengensis sp. n.

Taxon classificationAnimaliaLithobiomorphaLithobiidae

http://zoobank.org/C7382703-E3CE-445D-8166-6A3C34416C04

**Figure 1.**
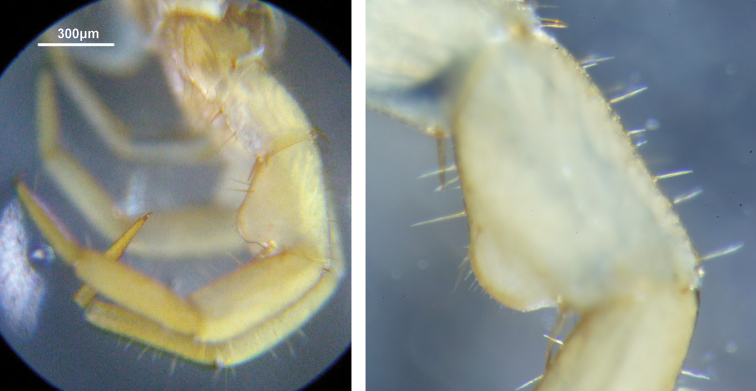
Lithobius (Monotarsobius) meifengensis sp. n., **A** ♂(NMNS7843-004), lateral view of male 15^th^ legs **B** ♂(NMNS7634-074), lateral view of male 15^th^ femur.

**Figure 2.**
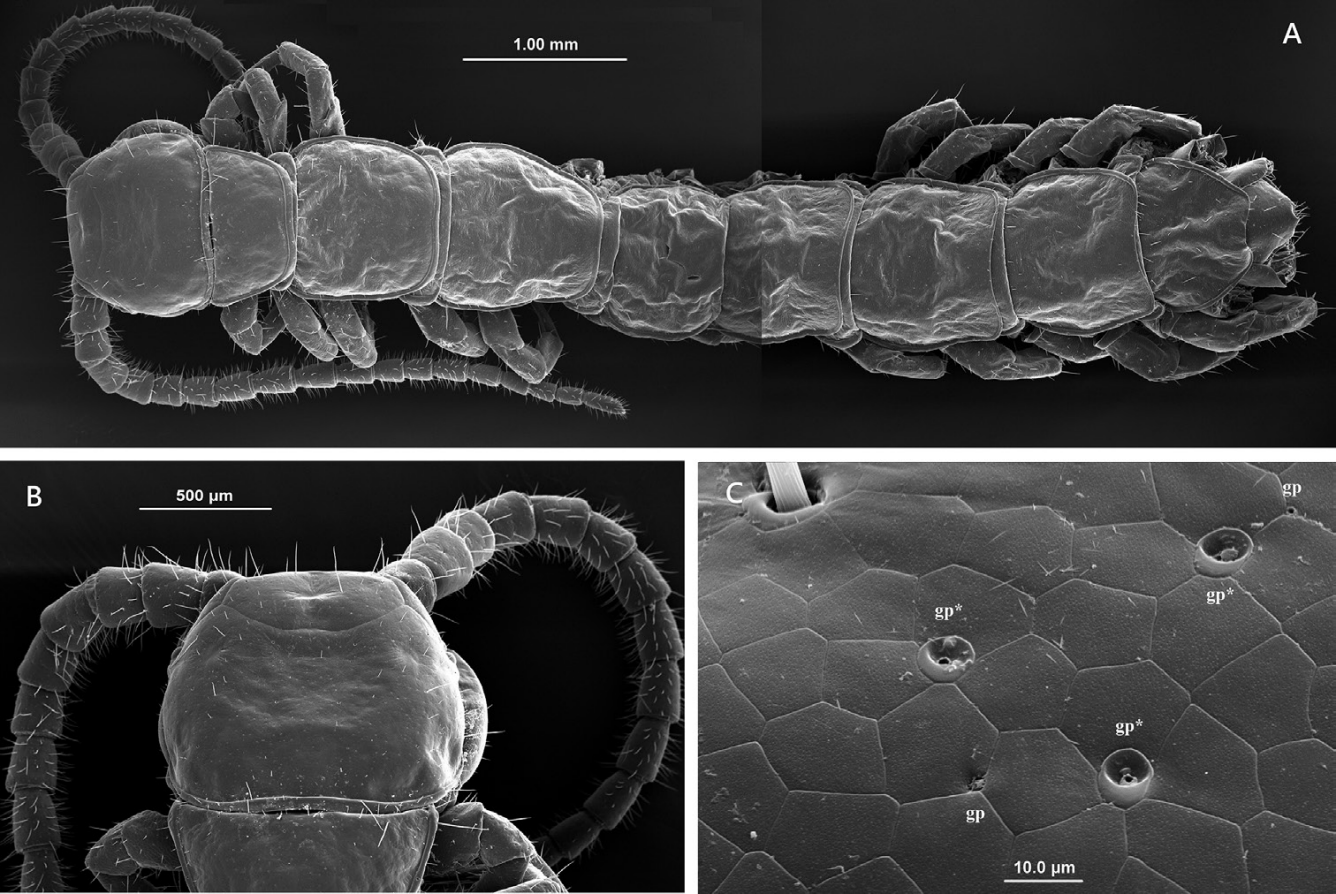
Lithobius (Monotarsobius) meifengensis sp. n. ♂ (NMNS7843-007), **A** habitus, dorsal view **B** cephalic plate **C** small pores of flexo-canal epidermal glands (gp) and large pores of recto-canal epidermal glands (gp*) on the cephalic plate.

**Figure 3.**
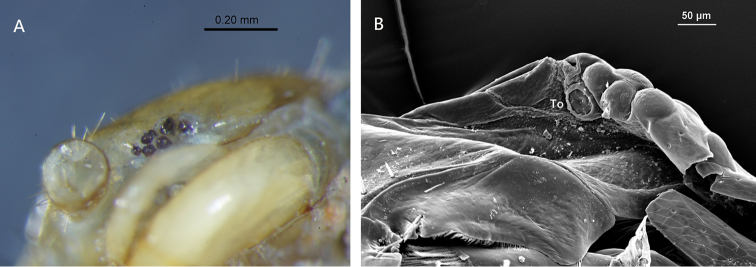
Lithobius (Monotarsobius) meifengensis sp. n. **A** ♂(NMNS7843-004), six ocelli on right side **B** ♂ (NMNS7843-008), Tömösváry’s organ (To).

**Figure 4.**
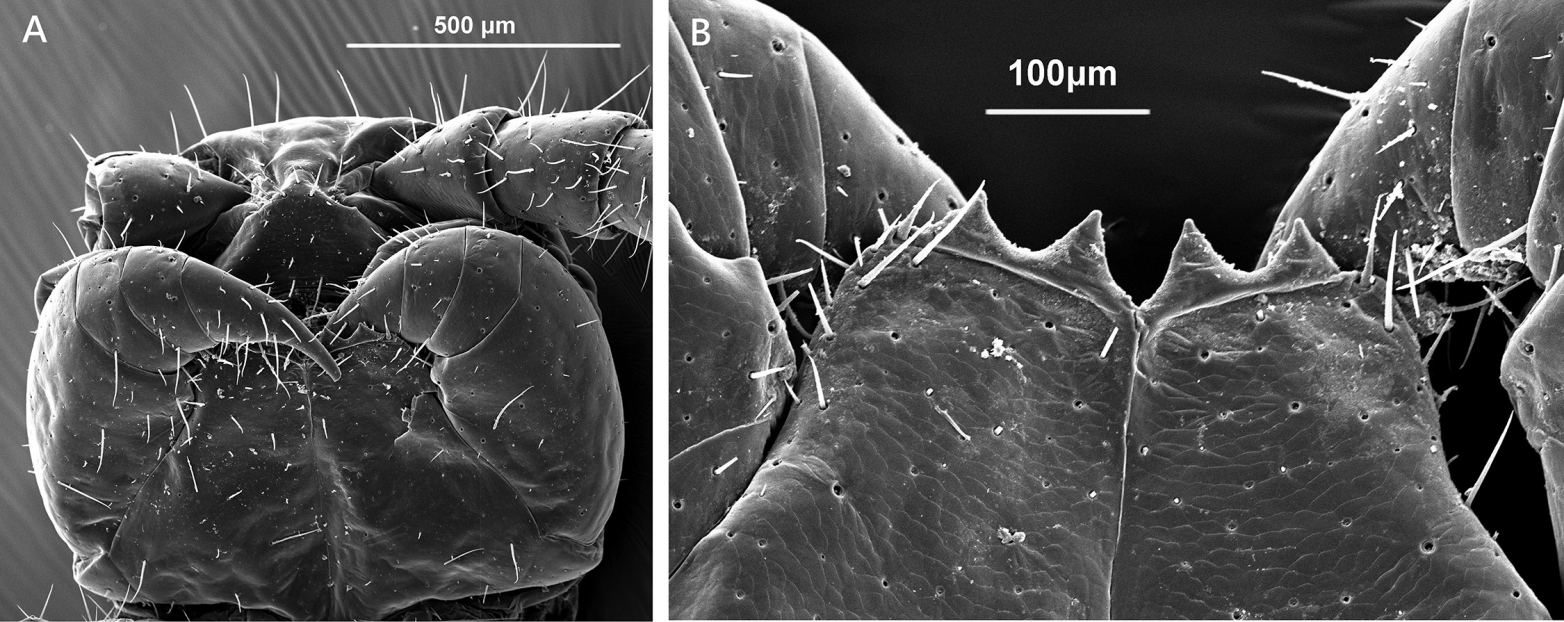
Lithobius (Monotarsobius) meifengensis sp. n., **A** ♂ (NMNS7634-070), ventral view of the head **B** ♂ (NMNS7843-008), coxosternal teeth and porodonts.

**Figure 5.**
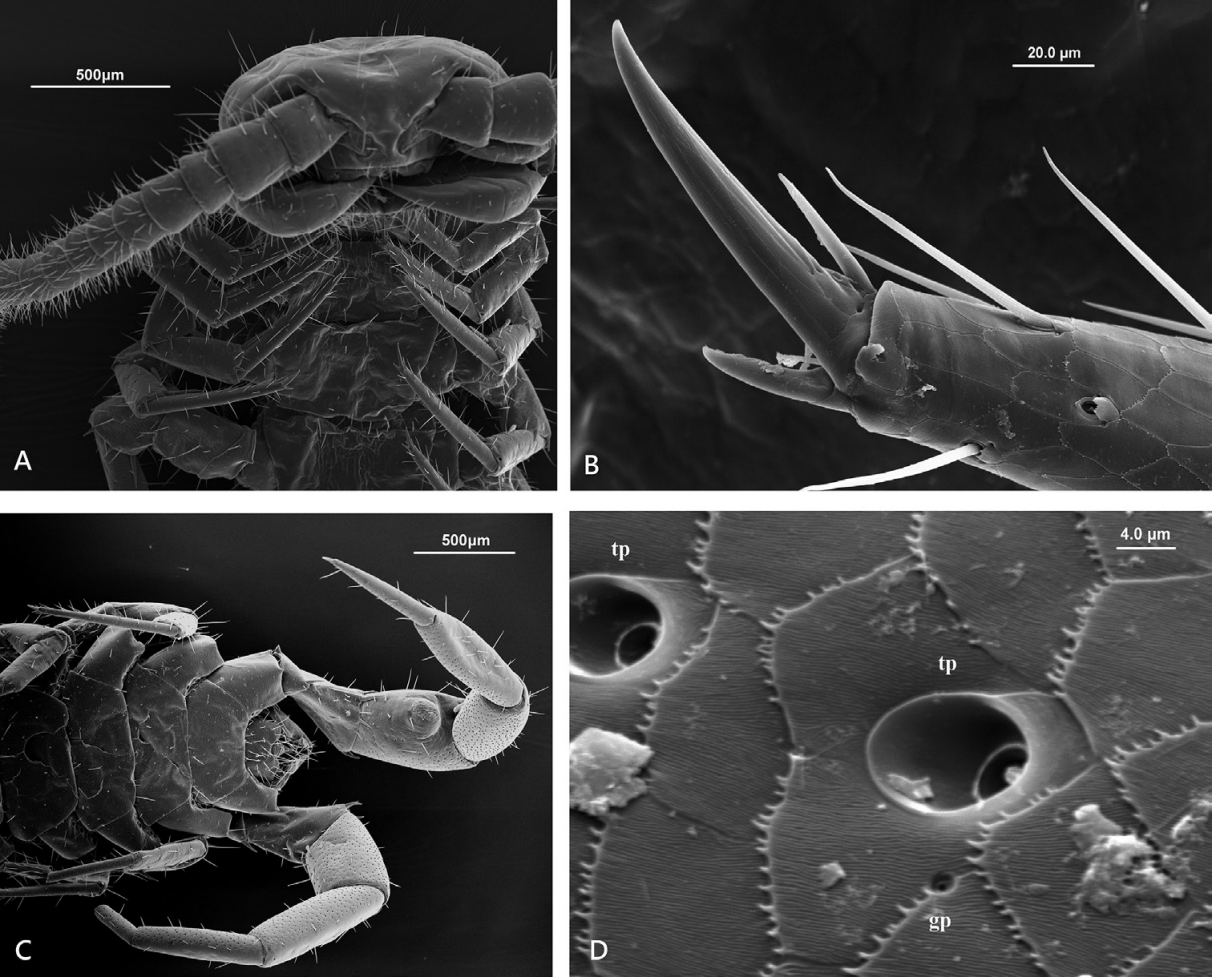
Lithobius (Monotarsobius) meifengensis sp. n. **A**, **B**, ♀NMNS7843-009: **A** the 1^st^ to 4^th^ leg-bearing segments, ventral view **B** the claw of 4^th^ leg **C, D** ♂ NMNS7634-070): **C** left 15^th^ leg and right 14^th^ leg, lateral-ventral view **D** large pores of the telopodal glands (tp) and small pore of flexo-canal epidermal gland (gp) on the 15^th^ leg.

**Figure 6.**
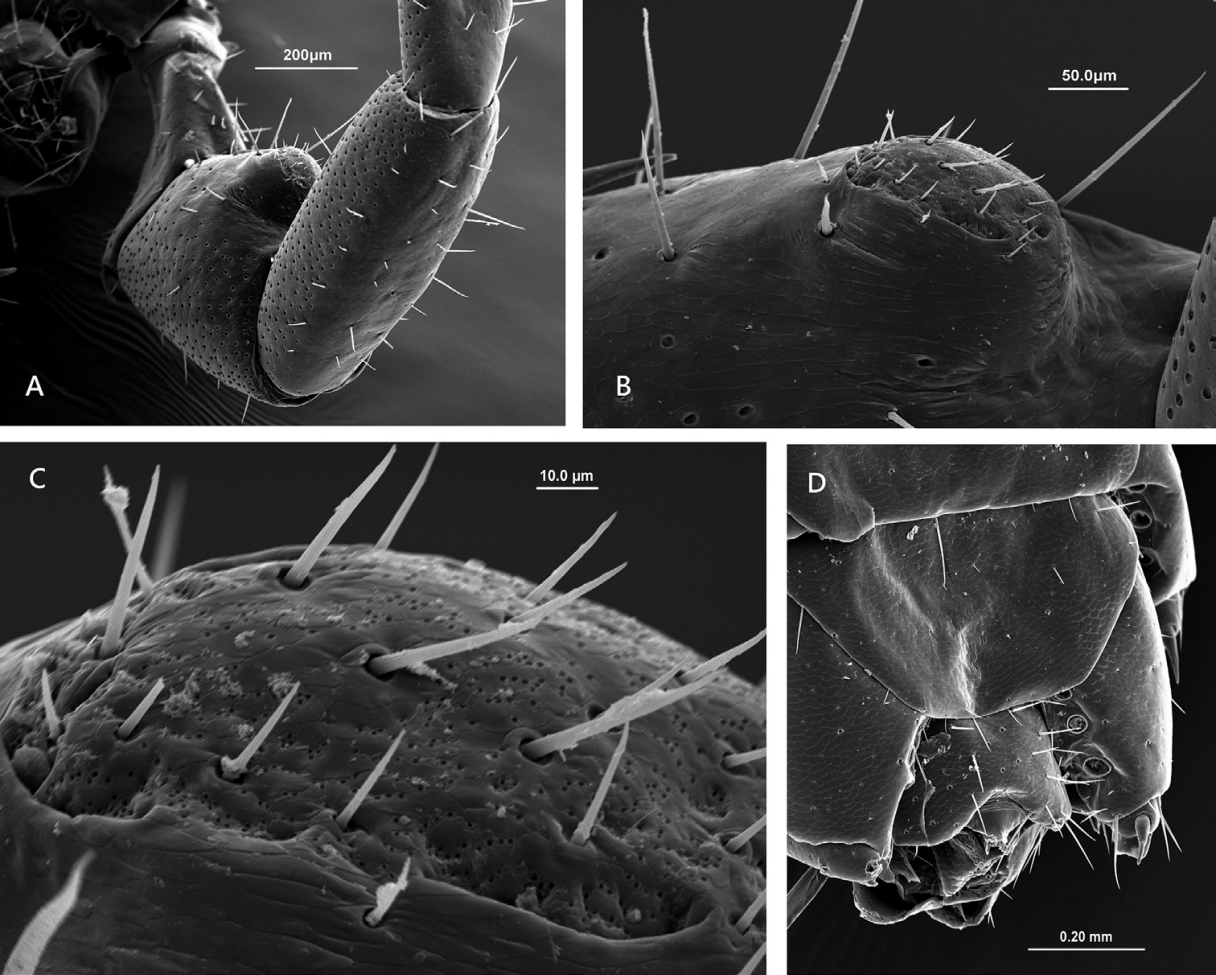
Lithobius (Monotarsobius) meifengensis sp. n. **A**–**C** ♂ NMNS7634-070): **A** the femur and tibia of male 15^th^ leg, ventral view **B** a large domed swelling on the ventral surface of male 15^th^ femur **C** apical region of the swelling on the male 15^th^ femur **D** ♂ NMNS7843-008, male genital sternite and 15^th^ sternite.

**Figure 7.**
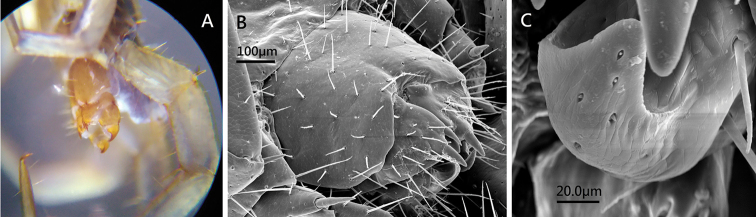
Lithobius (Monotarsobius) meifengensis sp. n. **A** (♀ NMNS7843-005): 15^th^ leg and female gonopod; **B**, **C** (♀ NMNS7843-009): **B** female genital sternite **C** terminal claw of female gonopod.

Figures 1 , 2 , 3 , 4 , 5 , 6 , 7

#### Type material.

**Holotype** ♂ (NMNS7634-073): Taiwan, Nantou County, Mei-Feng Farm, apple orchard, 24°05'N, 121°10'E, 2080 m, 19 Feb 2002, leg. Sheng-Hai Wu.

**Paratypes**:1♂ (NMNS7634-072), grassland; 1♂ (NMNS7634-074), plum orchard; 1♂ (NMNS7634-075); 1♀ (NMNS7634-068), pear orchard; 2♀ (NMNS7634-071), waste land; 1♂1♀ (NMNS7634-070), grassland; same data as holotype.

#### Other material.

1♀ (NMNS7634-096), pear orchard; 3♀ (NMNS7634-098), plum orchard; 1♂1♀ (NMNS7634-099), plum orchard; 1♂ (NMNS7634-100), pear orchard; 1♂4♀ (NMNS7843-006), grassland; 2♂1♀ (NMNS7843-007), grassland; 1♂2♀ (NMNS7843-003), grassland; 2♂5♀ (NMNS7843-004), plum orchard; 2♀ (NMNS7843-005), pear orchard; 2♀ (NMNS7843-002), grassland; 1♂ (NMNS7843-001), pear orchard; 2♂5♀ (NMNS7843-009), grassland; 2♂1♀ (NMNS7843-008), pear orchard; same locality as holotype, 15 Apr 2002, leg. Sheng-Hai Wu.

#### Etymology.

Refers to the type locality.

#### Diagnosis.

A species of the genus Lithobius Leach, 1814, subgenus Monotarsobius Verhoeff, 1905, normally with 19+19 elongate antennal articles, body length approximately 9 mm; cephalic plate 0.8–0.9 times as long as wide; six ocelli [one posterior and three dorsal, two ventral] on each side, posterior ocellus comparatively large; Tömösváry’s organ moderately small, slightly larger than adjacent ocelli; 2+2 coxosternal teeth; porodonts moderately slender, posterolateral to the outer tooth; posterior angles of all tergites lacking triangular projections; tarsi fused on legs 1–13; male secondary sexual characters on legs 15, a large ventral domed swelling on femur (Figure 1), and a dorsal shallow excavation on tarsus 2; coxal pores round, 3333 in males, 3443 or 3444 in females; female gonopods with 2+2 sharp coniform spurs, claw undivided.

#### Description.

Body length: 7.0–9.8 mm. Body colour (in alcohol): yellowish with dark patches.

*Antennae* with 19 articles (Figure 2A); basal three articles typically wider than long, following articles markedly longer than wide; distal article much longer than wide, up to 2.8 times as long as wide; abundant setae on antennal surface, less so on basal articles, gradual increase in density to around fourth article, then more or less constant in number.

*Cephalic plate* smooth, convex, 0.8–0.9 times as long as wide; posterior marginal ridge moderately broader and weakly concave (Figure 2B); small pores of flexo-canal epidermal glands (gp), large pores of recto-canal epidermal glands (gp*) and setae scattered sparsely over the whole surface (Figure 2C) (Müller, 2009).

Six *ocelli* on each side, one posterior and three dorsal, two ventral, arranged in two irregular row (Figure 3A); the posterior ocellus comparatively large; ocelli domed, translucent, usually darkly pigmented.

*Tömösváry’s organ* comparatively small, nearly rounded; situated at anterolateral margin of cephalic plate, slightly bigger than the adjoining ocelli (Figure 3B).

*Forcipular coxosternite* sub-trapezoidal, anterior margin narrow, external side lightly longer than internal side; median longitudinal cleft moderately deep (Figure 4A); anterior border with 2+2 large triangular coxosternal teeth, inner tooth slightly larger than outer one; porodonts moderately slender, setiform, posterolateral to the outer tooth (Figure 4B); some scattered setae on the ventral side of coxosternite.

*Tergites* smooth, without wrinkles, backside slightly hunched; T1 generally trapeziform, posterior margin narrower than anterior margin, narrower than T3 and the cephalic plate; T3 slightly narrower than the cephalic plate; posterior margin of TT1, 3, 5, 8, 10 and 12 weakly concave; TT1, 3 and 5 with continuous lateral and posterior marginal ridges, other tergites with discontinuous posterior marginal ridges; posterior angles of all tergites lacking triangular projections (Figure 2A); tiny setae scattered very sparsely over the surface.

*Sternites* narrower posteriorly, generally trapeziform, comparatively smooth, setae emerging from pores scattered very sparsely over the surface.

*Legs*: tarsi fused on legs 1–13 (Figure 5A), well-defined on legs 14–15; all legs with fairly long claws, curved ventrally; anterior and posterior accessory spines on legs 1–14, the anterior one moderately slender, the posterior spine short and thick (Figure 5B); legs15 lack anterior accessory spines; legs 14–15 with numerous large pores (9.1–11.1 μm) of the telopodal glands on the inner surfaces of femur, tibia, tarsus 1 and tarsus 2 (Figure 5C), the pores each opening into the centre of a bell-shaped cavity (3.5–3.9 μm) (Figure 5C), some small pores (1.4–1.6 μm) of flexo-canal epidermal glands sparsely distributed along the border of the epidermal cells (Figure 5D). Male 15^th^ legs with secondary sexual character; female legs 15 and other legs without secondary sexual characters on femur or tibia (Figure 7A). Leg plectrotaxy as in Table 1.

**Table 1.** Leg plectrotaxy of Lithobius (Monotarsobius) meifengensis sp. n.

leg	Ventral					Dorsal				
	C	t	P	F	Ti	C	t	P	F	Ti
1	–	–	–	am	m	–	–	p	ap	a
2	–	–	–	am	m	–	–	p	ap	a
3	–	–	–	am	m	–	–	p	ap	a
4	–	–	–	am	m	–	–	p	ap	a
5	–	–	–	am	m	–	–	p	ap	a
6	–	–	–	am	m	–	–	p	ap	ap
7	–	–	–	am	m	–	–	p	ap	ap
8	–	–	m	am	m	–	–	ap	ap	ap
9	–	–	m	am	m	–	–	ap	ap	ap
10	–	–	m	am	am	–	–	ap	ap	ap
11	–	–	mp	amp	am	–	–	ap	ap	ap
12	–	m	mp	amp	am	–	–	amp	ap	ap
13	–	m	mp	amp	am	–	–	amp	p	ap
14	–	m	mp	am	–	–	–	amp	–	–
15	–	m	amp	am	–	–	–	amp	–	–

Male secondary sexual character on leg 15: a large domed swelling on the ventral surface of femur, covering almost 50% (Figure 1, 6A); the surface of femoral swelling lacks the large pores of the telopodal glands (Figure 6B); the gently curved apical region bears approximately 20 short setae, and numerous very small pores (0.8–1.0 μm) of flexo-canal epidermal glands densely distributed (Figure 6C); a dorsal shallow excavation on the tarsus 2 (Figure 5C).

*Coxal pores*: 3333 in males, 3443 or 3444 in females, round, coxal pore field set in a relatively shallow groove, margin of coxal pore-field with slightly eminence.

*Male sternite* 15: trapeziform, posterolaterally narrower than anterolaterally, posterior margin straight, long setae scattered sparsely over the surface.

Male first genital sternite: wider than long, usually well chitinised; posterior margin quite deeply concave between the gonopods, without a medial bulge (Figure 6D); comparatively long setae evenly scattered on the ventral surface; gonopods short and small, with 2–3 long setae, apically slightly chitinized.

*Female sternite* 15: generally trapeziform, anterolaterally broader than posterolaterally, posterior margin straight, long setae scattered sparsely over the surface; the sternite of genital segment well chitinised, wider than long; posterior margin of genital sternite deeply concave (Figure 7B); short to long setae sparsely scattered over the ventral surface of the genital segment.

*Female gonopod*: first article fairly broad, bearing 11–13 long setae, arranged in three irregular rows; 2+2 sharp coniform spurs, inner spur smaller (Figure 7A, B); second article with 7–9 rather long setae arranged in two irregular rows on its ventral side; third article usually with 2–3 long setae on its ventral surface; terminal claw undivided, bearing a few thick sensilla coeloconica on its dorsodistal and ventral surface (Figure 7C).

##### Remarks

Some *Lithobius* species, all from Russia, also have distinct sexual characters on male leg 15: the proximal part of male 15^th^ tibia of Lithobius (Monotarsobius) kurcheavae described by Zalesskaja (1978) has a large swelling bearing a tuft of long bristles, and a longitudinal deep excavation on the dorsal surface; the dorsal surface of the male 15^th^ tibia of Lithobius (Monotarsobius) evsyukovi (Zuev 2017) a large, flat, ovoid wart supporting a few short setae at apex; the dorsal surface of 15^th^ tibia Lithobius (Monotarsobius) ferganensis (Trotzina, 1894) a small cylindrical wart supporting a few short setae at apex; while the male 15^th^ tibia of Lithobius (Chinobius) yuchernovi (Farzalieva et al. 2017) is characterized by a tubercle supporting a cluster of curved and long setae on the ventral surface. Lithobius (Monotarsobius) meifengensis sp. n. differs from those by its unique male secondary sexual character on the ventral surface of 15^th^ femur, a large swelling with approximately 20 short setae and numerous very small pores, not found in any of those congeners.

Records of the species of Lithobius (Monotarsobius) from Taiwan by Takakuwa and Wang are listed as follows: locality: old name = new name; place name ??: unknown.

Lithobius (Monotarsobius) ramulosus (Takakuwa, 1941)

[1] *Monotarsobius ramulosus* Takakuwa, 1941a – Trans. Nat. Hist. Soc. Formosa 31 (213): 294-295; fig. 5, 6 (original description, key) (locality: Keisyu = Xizhou)

*Monotarsobius ramulosus*: Takakuwa, 1941b – Fauna Nippon. 9(8-3): 74; fig. 84; (description, key) (locality: Keisyu = Xizhou)

*Monotarsobius ramulosus*: Takakuwa, 1942 –Trans. Nat. Hist. Soc. Formosa 32(231): 360 (locality: Keisyu = Xizhou)

*Monotarsobius ramulosus*: Wang 1955 – Quar. J. Taiwan Mus. 8(1): 16 (locality: Taipei)

*Monotarsobius ramulosus*: Wang 1956 – Quar. J. Taiwan Mus. 9(2): 159 (locality: Hualien)

Lithobius (Monotarsobius) obtusus (Takakuwa, 1941)

[1] *Monotarsobius obtusus* Takakuwa, 1941a – Trans. Nat. Hist. Soc. Formosa 31 (213): 293-294; fig. 2 (original description, key) (locality: Keisyu = Xizhou, Shaka = Shalu, Tikunan = Zhunan)

*Monotarsobius obtusus*: Takakuwa 1941b – Fauna Nippon. 9(8-3): 75; fig. 85; (description, key) (locality: Keisyu = Xizhou, Shaka = Shalu)

*Monotarsobius obtusus*: Wang 1955 – Quar. J. Taiwan Mus. 8(1): 16 (locality: Shin-Tien = Hsintien)

*Monotarsobius obtusus*: Wang 1956 – Quar. J. Taiwan Mus. 9(2): 159 (locality: Hualien)

*Monotarsobius obtusus*: Wang 1957 – Quar. J. Taiwan Mus. 10(1): 28 (locality: Kao Yung ??)

*Monotarsobius obtusus*: Wang 1959 – Quar. J. Taiwan Mus. 12(3, 4): 198 (locality: Taipei, Kao Yung ??)

*Monotarsobius obtusus*: Wang 1963 – Quar. J. Taiwan Mus. 16(1, 2): 95 (locality: Rai Wu ??)

Lithobius (Monotarsobius) holstii (Pocock, 1895)

[1] *Monotarsobius crassipes holstii* (+*M. takakuwai*): Takakuwa 1941a – Trans. Nat. Hist. Soc. Formosa 31 (213): 292-293; fig. 1(description, key)

*Monotarsobius crassipes holstii* (+*M. takakuwai*): Takakuwa 1941b – Fauna Nippon. 9 (8-3): 78-79; fig. 90-91; (description, key) (locality: Puli, Taipei)

[2] *Monotarsobius crassipes*: Wang 1959 – Quar. J. Taiwan Mus. 12 (3, 4): 198 (locality: Nantou, Taipei)

*Monotarsobius crassipes*: Wang 1963 – Quar. J. Taiwan Mus. 16 (1, 2): 95 (locality: Shao Tso Kiang ??)

Chamberlin and Wang (1952) recorded two species of *Monotarsobius* from three specimens allegedly collected by Takakuwa in 1933 from Taiwan: *Monotarsobius rhysus* Attems, 1934 and *Monotarsobius argaeensis* Attems, 1934. However, Takakuwa never came to Taiwan, and never reported these two species in any of his publications. Wang came to Taiwan and studied Taiwanese chilopods since 1953, and he did not record these two species again. We consider that the record of the two species is questionable.

Lithobius (Monotarsobius) meifengensis sp. n. is morphologically close to Lithobius (Monotarsobius) ramulosus (Takakuwa, 1941), Lithobius (Monotarsobius) obtusus (Takakuwa, 1941) and Lithobius (Monotarsobius) holstii (Pocock, 1895), with which it shares the following characters: antennae composed of 19-20 articles, six ocelli on each side of cephalic plate, 2+2 coxosternal teeth. It can however be distinguished using the following key.

##### Key to the Taiwanese species of Lithobius (Monotarsobius)

**Table:** 

1	2222 coxal pores; terminal claw of female gonopod divided, biapiculate	***L. (M.) obtusus* Takakuwa, 1941**
–	3-5 coxal pores; terminal claw of female gonopod undivided	**2**
2	5555 coxal pores; a small sharp tooth on the base of terminal claw of female gonopod	***L. (M.) ramulosus* Takakuwa, 1941**
–	3-4 coxal pores; base of terminal claw of female gonopod without sharp tooth	**3**
3	Male legs 15 with secondary sexual characters, a large ventral swelling on the femur, a dorsal shallow excavation on the tarsus 2 (Figure 5C); terminal claw of female gonopod with smooth lateral margin, without ridge	***L. (M.) meifengensis* sp . n**
–	Male legs 15 without secondary sexual characters; terminal claw of female gonopod with irregular internal and external ridges	**L. (M.) holstii (Pocock, 1895)**

## Supplementary Material

XML Treatment for Lithobius (Monotarsobius) meifengensis
